# Hand Hygiene Education Components Among First-Year Nursing Students: A Cluster Randomized Clinical Trial

**DOI:** 10.1001/jamanetworkopen.2024.13835

**Published:** 2024-06-13

**Authors:** Jing Chen, Lin Yang, Yim-Wah Mak, Margaret O’Donoghue, Chen Shi, Hilda Tsang, Shuya Lu, Jing Zou, Jing Qin, Yao Jie Xie, Timothy Lai, Chen Li, Jiannong Cao, Didier Pittet

**Affiliations:** 1 School of Nursing, The Hong Kong Polytechnic University, Hong Kong SAR, China; 2 The Jockey Club School of Public Health and Primary Care, Chinese University of Hong Kong, Hong Kong SAR, China; 3 Research Centre of Textiles for Future Fashion, The Hong Kong Polytechnic University, Hong Kong SAR, China; 4 Joint Research Centre for Primary Health Care, The Hong Kong Polytechnic University, Hong Kong SAR, China; 5 School of Public Health, Southern Medical University, Guangzhou, China; 6 Department of Applied Social Science, The Hong Kong Polytechnic University, Hong Kong SAR, China; 7 Department of Computing, The Hong Kong Polytechnic University, Hong Kong SAR, China; 8 Faculty of Medicine, University of Geneva, Geneva, Switzerland

## Abstract

**Importance:**

Few studies have directly and objectively measured the individual and combined effects of multifaceted hand hygiene education programs.

**Objective:**

To evaluate the individual and combined immediate effects of an instructional video and hand scan images on handwashing quality, decontamination, and knowledge improvement.

**Design, Setting, and Participants:**

This cluster randomized clinical trial was conducted in June to July 2023 among first-year nursing students at a university in Hong Kong. The study used an intention-to-treat analysis.

**Intervention:**

Hand hygiene education sessions featuring an instructional video, hand scan images, or both.

**Main Outcomes and Measures:**

The primary outcome was the change in residue from fluorescent lotion remaining on participants’ hands after handwashing before and after the intervention. The secondary outcomes included handwashing quality and knowledge of hand hygiene.

**Results:**

A total of 270 of 280 students (mean [SD] age, 19 [1] years; 182 [67.4%] female) participated in the trial (96.4% participation rate). Participants were randomized to a control group (66 participants), hand scan image group (68 participants), instructional video group (67 participants), and hand scan image with instructional video group (69 participants). All intervention groups had greater reductions in residue after the intervention compared with the control group, although none reached statistical significance (hand scan image group: 3.9 [95% CI, 2.0-5.8] percentage points; instructional video group: 4.8 [95% CI, 2.9-6.7] percentage points; hand scan image with instructional video: 3.5 [95% CI, 1.6-5.4] percentage points; control group: 3.2 [95% CI, 1.3-5.2] percentage points). The instructional video group showed a significant improvement in their handwashing performance, with a higher percentage of participants correctly performing all 7 steps compared with the control group (22.4% [95% CI, 13.1% to 31.6%] vs 1.5% [−7.9% to 10.9%]; *P* < .001). Hand scan images revealed that wrists, fingertips, and finger webs were the most commonly ignored areas in handwashing.

**Conclusions and Relevance:**

In this cluster randomized clinical trial of an education program for hand hygiene, a handwashing instructional video and hand scan images did not enhance the level of decontamination. The intervention group had improved handwashing techniques compared with the control group, a secondary outcome.

**Trial Registration:**

ClinicalTrials.gov Identifier: NCT05872581

## Key Points

**Question:**

Which components of an educational program featuring a handwashing instructional video and hand scan images are effective at increasing the level of decontamination after handwashing among nursing students?

**Findings:**

This cluster randomized clinical trial involved 270 first-year nursing students and found that all groups, including the control group, had significantly less fluorescence residue on hands after the intervention. The groups that had access to the instructional video showed a significant improvement in handwashing performance in terms of the percentage of correctly performed handwashing steps compared with the control group.

**Meaning:**

In this study, a program that included an instructional video did not improve the amount of contaminants removed.

## Teaser

This cluster randomized clinical trial examines the individual and combined effects of components of a hand hygiene training program—an instructional video and hand scan images—on the level of decontamination after washing hands among first-year nursing students in Hong Kong.

## Introduction

Health care–associated infections (HAIs) pose a significant threat to patient safety and contribute to a substantial global burden of disease and mortality.^1^ One essential and cost-effective way to reduce HAIs is through proper hand hygiene. The World Health Organization (WHO) emphasizes that hand hygiene can decrease the incidence of HAIs by 70% and significantly reduce health care costs.^2^ The COVID-19 pandemic has highlighted the importance of hand hygiene in preventing disease transmission in both community and health care settings.^3,4^ Therefore, it is crucial to provide education and training programs for health care workers, who play a critical role in preventing HAIs.

Traditional hand hygiene training for health care workers often includes classroom lectures and practical sessions, but these can be constrained by costs and resources. Multifaceted interventions have proven to be the most effective in changing hand hygiene behavior.^5^ Following the successful model of a multimodal promotion strategy that significantly improved hand hygiene compliance and reduced both HAIs and cross-transmission rates of multiresistant organisms at the University of Geneva Hospitals in the late 1990s,^6^ the WHO developed *Guidelines for Hand Hygiene in Health Care*.^7^ This implementation strategy is now used in numerous institutions, regions, and countries worldwide.^7,8,9,10^

Previous studies have used various approaches to improve hand hygiene compliance in health care settings, such as self-assessment questionnaires and observation by trained individuals.^11,12^ Educational programs that incorporate monitoring and feedback can positively influence hand hygiene compliance.^12,13^ Various methods, such as posters, videos, and role-playing, have been adopted.^14^ However, further research is needed to evaluate the individual effects of each component of these training program. There is a lack of research that has directly and objectively measured the impact of these programs on hand hygiene techniques and the quality of performance. There is also a research gap in the understanding of the efficacy of different hand hygiene techniques. A systematic review^15^ found inconclusive results regarding whether the WHO 6-step technique is superior to alternatives such as the US Centers for Disease Control and Prevention’s 3-step and adapted 6-step techniques. The 7-step hand hygiene technique is currently adopted in some countries and regions, including Hong Kong and mainland China.^16^

While nursing education acknowledges the importance of hand hygiene in preventing HAIs, research indicates that nursing students often have only low to moderate knowledge and compliance.^17^ The use of technologies, such as electronic monitoring systems and gaming programs, could potentially enhance student-focused approaches, promoting compliance and improving overall hand hygiene practices.^18^ Implementing strategies including feedback mechanisms, improving clinical conditions, and using checklists and protocols are crucial for good hand hygiene practices.

In this study, we developed an educational program consisting of a handwashing instructional video along with hand scan images that allow students to visually identify overlooked areas during handwashing. We conducted a cluster randomized clinical trial among first-year nursing students to assess the immediate effects of the individual components and the combined program on decontamination as well as handwashing quality and knowledge.

## Methods

### Study Design

We conducted a cluster randomized clinical trial, with each cluster representing a single hand hygiene training session. This design was adopted to address difficulties associated with implementing distinct interventions for individually randomized nursing students and potential cross-contamination.^19,20^ The trial was reviewed and approved by the institutional review board of Hong Kong Polytechnic University. Before enrolment, all participants gave written informed consent. The trial protocol (Supplement 1) remained unchanged throughout the trial. This trial was conducted in line with the Declaration of Helsinki and adhered to the Consolidated Standards of Reporting Trials (CONSORT) reporting guideline.^21^

### Participant Recruitment and Randomization

From June 1 to July 31, 2023, we invited all first-year nursing students from the School of Nursing at the Hong Kong Polytechnic University to participate in our study. Before the trial, students attended a lecture on the importance of hand hygiene, which did not include instructional videos or detailed handwashing techniques. We then asked participants to register for a 1-on-1 training session lasting 15 to 20 minutes, arranged in half-day clusters with a maximum of 10 participants each. A statistician not involved in recruiting the participants generated random numbers for group formation. Participants were not informed of their group assignments until they attended their designated sessions. The lecture instructor and experts evaluating hand hygiene quality from video recordings were masked to group assignments. However, the research staff responsible for data collection at the study site were informed, as they provided instructions to participants.

### Sample Size Estimation

To estimate the sample size for this study, we made several assumptions based on the results of our previous study.^22^ We assumed a standard deviation of 2.00 for individual participants, an intracluster correlation coefficient of 0.01, and a coefficient of variation of 0.2 for cluster sizes. With a sample size of 280, we aimed to achieve a statistical power of 0.8. This would allow us to detect a minimum difference in the percentage of residual florescence of at least 1 in the means between the groups after the intervention, using a 2-sided *t* test with a significance level of .05.^23^

### Data Collection

Before the intervention, participants completed a questionnaire that collected sociodemographic information and their past experience with hand hygiene training. They then applied a fluorescent lotion to their hands, ensuring it covered their entire hands, and had their hands scanned without contact to verify the coverage. A research assistant guided and recorded the participants as they washed their hands with liquid soap at a sink. One pump of liquid soap was dispensed to maintain the consistency in soap volume, which might affect the effectiveness of handwashing.^24^ The participants were randomly divided into 4 groups: (1) the hand scan image group, who received immediate feedback from the images of their hand scans; (2) the instructional video group, who watched an instructional video that demonstrated the 7-step handwashing process according to local guidelines^16^; (3) the hand scan image with instructional video group, who received visual feedback and watched the instructional video; and (4) the control group, who did not receive any feedback or watch any video. The first 3 groups underwent interventions after their first handwashing attempt, while the control group watched the video after the intervention measurements were taken. A red-green-blue (RGB) camera was used to record the hand movements of the participants during handwashing, and infection prevention experts evaluated their performance based on the length of each step and the percentage of handwashing steps performed correctly. More details of the trial procedure are described in the eMethods in Supplement 2.

### Outcome Measurements

The primary outcome of the study was the change in the residue from fluorescent lotion remaining on the palms and backs of participants’ right and left hands after handwashing before and after the intervention. The hand scanner used ultraviolet light to highlight fluorescent residues on hands, and a built-in camera captured images, visualizing areas with residue. The system used image analysis software to accurately measure residue coverage.^25^ Secondary outcomes included the quality of handwashing that was assessed through video recordings of the 7-step handwashing process and participants’ knowledge of hand hygiene collected through a questionnaire. Once the trial began, no changes were made to these predetermined outcomes. All assessments were conducted before and after the intervention.

### Statistical Analysis

The statistical analysis was performed using Stata version 18.0 (StataCorp) and SAS version 9.4 (SAS Institute) with an intention-to-treat approach, considering *P* < .05 statistically significant. Participants with missing data on the percentage of correctly performed handwashing steps (<0.07%) were excluded from the analysis. Kruskal-Wallis test and the χ^2^ test were used to compare continuous (eg, percentage of fluorescent residue) and categorical variables (eg, step correctness) across groups. The Dunn test was used for nonparametric pairwise multiple comparisons. Wilcoxon signed rank test was used to test the difference in repeated measurements within each group.

Multilevel analyses accounted for the repeated measure in primary and secondary outcomes taken before and after the intervention were performed. The difference between the intervention and control clusters in pre-post changes in primary and secondary outcomes was estimated using a mixed model (Stata command mixed) with a random intercept of cluster. The model included fixed effects for group, time (before and after the intervention), group × time interaction, and baseline characteristics including sex, age, program enrolled, and previous training. Time was specified within each participant to address repeated measurements in outcomes over time. A subgroup analysis was conducted for both the palm and back of the left and right hands.

To visualize the hand areas with higher concentrations of fluorescent residue, we merged the hand image files for each group using advanced machine learning techniques. More information about this technology can be found in our previous study.^22^

## Results

### Participant Characteristics

**Figure 1.**
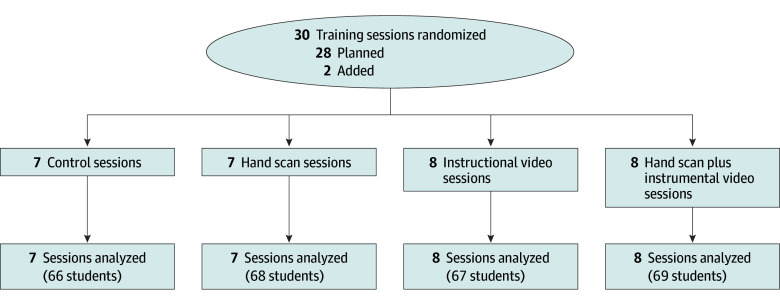
Trial Flowchart

Figure 1 shows a flowchart of the trial. A total of 270 of 280 first-year nursing students, with a mean (SD) age of 19 (1) years and a participation rate of 96.4%, were divided into 30 training clusters. These clusters were then randomly assigned to 1 of 4 study groups by a statistician, with each cluster containing between 6 and 12 participants.

Most of the participants were female (182 [67.4%]), and 50 (18.7%) reported having received hand hygiene training in the past. The characteristics of the participants and the proportion of those who had received any formal hand hygiene training were similar across all 4 groups (Table 1).

**Table 1.** Baseline Characteristics of the Participating First-Year Nursing Students

Characteristics	Participants, No. (%)			
	Control (n = 66)	Hand scan image (n = 68)	Instructional video (n = 67)	Hand scan image with instructional video (n = 69)
Age, median (IQR), y	19 (18-22)	18 (18-22)	19 (18-21)	18 (18-21)
Sex, female				
Female	42 (63.6)	46 (67.6)	47 (70.1)	47 (68.1)
Male	24 (36.4)	22 (32.4)	20 (29.1)	22 (31.9)
Bachelor program				
Nursing	51 (77.3)	51 (75.0)	49 (73.1)	48 (69.6)
Mental health nursing	15 (22.7)	17 (25.0)	18 (26.9)	21 (30.4)
Previous formal training on hand hygiene	15 (22.7)	14 (20.6)	8 (12.1)	13 (19.1)

### Primary Outcome

Before the intervention, the percentage of fluorescent lotion residue, which was measured as a percentage from 0 to 100% (with higher percentages indicating worse results), did not significantly vary among groups. It ranged from 5.0% (95% CI, 2.9%-7.2%) in the hand scan image with instructional video group to 6.6% (95% CI, 4.5%-8.7%) in the instructional video group (Table 2). After the intervention, all groups showed a significant decrease in fluorescence residue (absolute difference in percentage: hand scan image group, 3.9 [95% CI, 2.0-5.8] percentage points; instructional video group, 4.8 [95% CI, 2.9-6.7] percentage points; hand scan image with instructional video group, 3.5 [95% CI, 1.6-5.4] percentage points; control group, 3.2 [95% CI, 1.3-5.2] percentage points). After adjusting for baseline characteristics and preintervention measures, no significant difference in the changes in fluorescence residues before and after the intervention was found in the intervention groups compared with the control group.

**Table 2.** Handwashing Effectiveness Measured by Hand Scanner

Hand area	Residue, % (95% CI)				ICC (SE) [95% CI]^a^
	Control (n = 66)	Hand scan image (n = 68)	Instructional video (n = 67)	Hand scan image with instructional video (n = 69)	
Both hands					
Before intervention	5.90 (3.73-8.07)	5.24 (3.11-7.37)	6.60 (4.46-8.74)	5.04 (2.93-7.15)	0 (0.03) [0.0-0.06]
After intervention	2.66 (1.72-3.60)	1.35 (0.43-2.27)	1.76 (0.85-2.68)	1.55 (0.64-2.46)	0.04 (0.04) [0.0-0.11]
*P* value^b^	NA	.63	.23	.83	NA
Palm of left hand					
Before intervention	5.79 (3.72-7.86)	5.35 (3.32-7.38)	6.47 (4.43-8.51)	4.58 (2.57-6.59)	0 (0.03) [0.0-0.06]
After intervention	2.72 (1.56-3.87)	1.54 (0.41-2.67)	2.17 (1.05-3.30)	1.71 (0.60-2.82)	0.02 (0.03) [0.0-0.09]
* P* value^b^	NA	.58	.33	.90	NA
Palm of right hand					
Before intervention	7.47 (4.73-10.21)	6.85 (4.17-9.54)	9.34 (6.64-12.03)	6.15 (3.49-8.81)	0.01 (0.03) [0.0-0.07]
After intervention	3.80 (2.34-5.25)	1.93 (0.49-3.36)	2.35 (0.92-3.78)	2.25 (0.84-3.66)	0.03 (0.04) [0.0-0.11]
* P* value^b^	NA	.47	.05	.87	NA
Back of left hand					
Before intervention	5.08 (2.57-7.59)	3.83 (1.36-6.30)	5.37 (2.88-7.85)	4.73 (2.28-7.18)	0 (0.03) [0.0-0.06]
After intervention	2.08 (1.17-2.99)	1.05 (0.15-1.94)	1.09 (0.20-1.99)	1.28 (0.40-2.16)	0.02 (0.04) [0.0-0.09]
*P*value^b^	NA	.89	.42	.76	NA
Back of right hand					
Before intervention	5.29 (2.90-7.68)	4.94 (2.59-7.29)	5.22 (2.85-7.59)	4.76 (2.42-7.09)	0.01 (0.03) [0.0-0.07]
After intervention	2.09 (1.25-2.92)	0.88 (0.06-1.70)	1.44 (0.63-2.26)	1.00 (0.19-1.81)	0.03 (0.04) [0.0-0.10]
*P* value^b^	NA	.60	.70	.71	NA

Abbreviations: ICC, intracluster correlation coefficient; NA, not applicable.

^a^ Negative values have been truncated by loneway command in Stata.

^b^ Difference in changes before and after intervention, each intervention group compared with the control group. The model adjusted for baseline age, sex, program attended, previous hand hygiene training experience, and preintervention measures.

### Secondary Outcomes

Accuracy and duration of each handwashing step was assessed by our infection prevention and control experts using video recordings. Before the intervention, the duration of each step ranged from 3 to 9 seconds (eTable 1 in Supplement 2), with all groups showing significant increases after the intervention. The total duration of the 6- and 7-steps, per WHO^7^ and local^16^ guidelines, increased by 12 to 13 seconds and 15 to 16 seconds, respectively, which was much longer than the control group’s increase (6 and 7 seconds). Less than 18% of participants correctly performed all 7 steps, with the instructional video group and the hand scan image with instructional video group showing significant improvements compared with the control group (eTable 1 in Supplement 2). Apart from the instructional video group (which increased by 22.4% [95% CI, 13.1%-31.6%] vs 1.5% [−7.9% to 10.9%]; *P* < .001), there were no significant differences among the 4 groups in terms of the percentage of participants who performed all 7 steps correctly. In general, the quality and duration of handwashing for all 6- or 7-steps were similar among the intervention groups but were better and longer than the control group.

Before the intervention, participants had a moderate knowledge level of handwashing steps, types, minimum duration, and sequence (eTable 2 in Supplement 2). The instructional video group demonstrated significant improvements in correctly answering questions about hand hygiene steps (from 56.1% to 70.1%; *P* = .03) and sequence (from 57.6% to 76.1%; *P* = .01) in the preintervention and postintervention questionnaires. After the intervention, attitudes toward hand hygiene improved across all groups, with a significant increase in the number of individuals strongly agreeing that “hand hygiene is part of infection prevention and control in healthcare settings” (eTable 3 in Supplement 2). There was a significant improvement in the duration and accuracy of each step (except step 1 and 7) in the intervention groups compared with the control group (eFigure in Supplement 2). The instructional video group showed greater improvements in knowledge compared with the control group (eTable 2 in Supplement 2).

**Figure 2.**
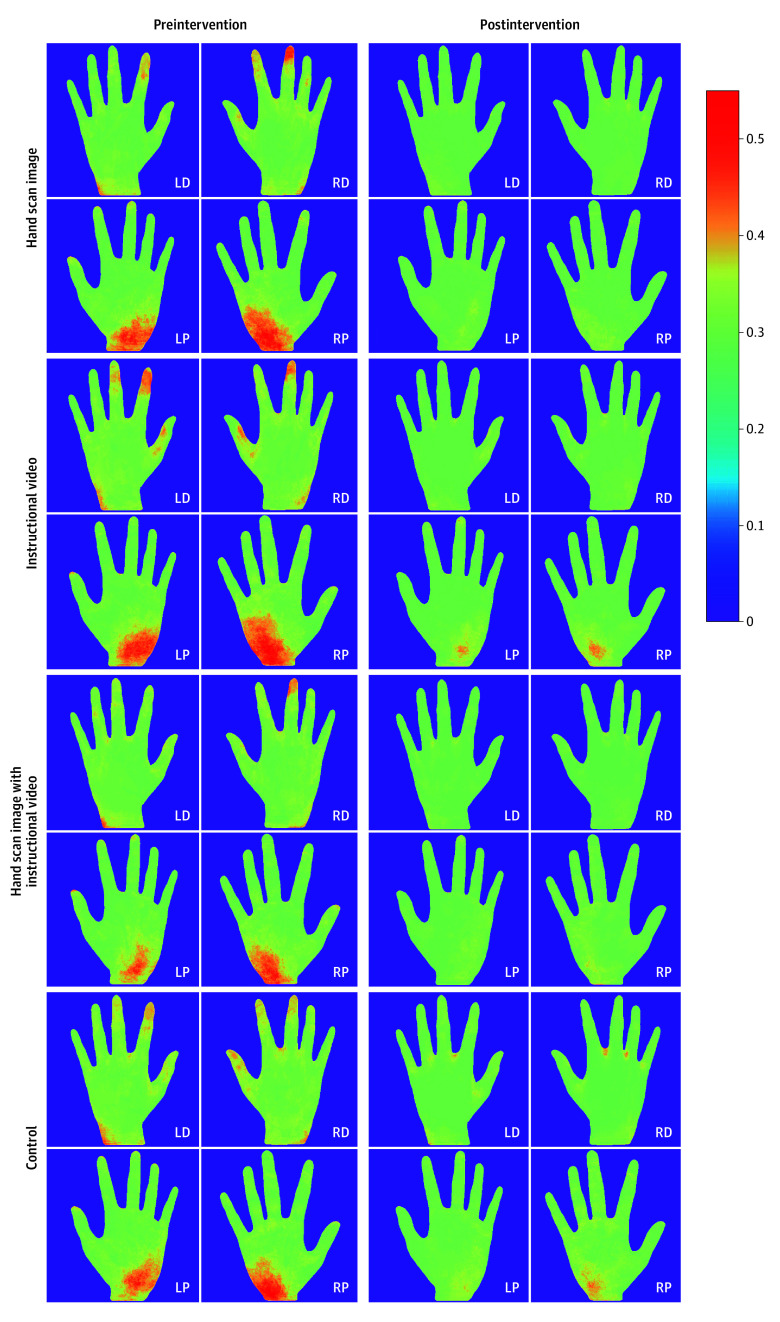
Overlaid Color Map Images of Palm and Dorsum of Left and Right Hands After Normalization The color ranges from 0 (no fluorescent residual) to 1 (maximum fluorescent residual). LD indicates left dorsum; LP, left palm; RD, right dorsum; RP, right palm.

Figure 2 shows the merged hand scan images from approximately 2000 hand images that were processed and registered using advanced normalization algorithms. Each group had between 248 and 264 images, with 4 images per participant. Before the intervention, the palms of both hands had less fluorescent residue than the dorsum, with the wrists, fingertips, and finger webs being the most common areas with residual fluorescence. All intervention groups showed greater reductions in fluorescence residues after the intervention, compared to the control group.

## Discussion

To our knowledge, this is the first intervention study to evaluate the individual and combined effects of an instructional video and hand scan images on the quality of handwashing and knowledge of hand hygiene. We adopted objective measures to assess the quality of handwashing and the effect of decontamination as well as comprehensive evaluations of the accuracy and duration of each step. We found that both the instructional video and hand scan images significantly improved the quality of handwashing techniques. After the intervention, all intervention groups showed improvements in removing contaminants. However, the relative change of decontamination effects was not significantly different from the control group. Although combining these 2 components did not provide additional benefits in terms of removing fluorescence residues, each contributed uniquely to enhancing different aspects of handwashing quality. Visual feedback had a greater impact on improving handwashing quality, while the instructional video primarily affected technique. This is supported by the findings that the percentage of correctly performed handwashing steps improved more in the instructional video group and the hand scan image with instructional video group, but not in the hand scan image group compared with the control group. Our findings further support the WHO’s recommendations of using a multifaceted education program to promote good hand hygiene.^7^

The Clean Care Is Safer Care campaign, launched by the WHO in 2005, and the subsequent Save Lives: Clean Your Hands program set hand hygiene standards worldwide.^1,7^ Our education program used a 1-minute instructional video that followed the 7-step technique according to local guidelines^16^ and found it effective in improving participants’ knowledge and practice. Particularly, we found that the instructional video significantly improved the performance quality compared with the control group, consistent with previous studies that demonstrated the effectiveness of video-assisted teaching in enhancing hand hygiene compliance and accuracy.^26^

Our study echoed previous findings that the effective hand hygiene involves both knowledge enhancement and practice improvement.^27,28^ The utilization of hand scan images had a positive impact on improving the handwashing quality of nursing students and increasing their awareness of previously neglected hand areas during hand rubbing.^29^ A previous study in frontline health care workers found that the hand scanner significantly improved hand hygiene techniques and compliance rates by providing immediate feedback.^30^ Our study also found that hand scan images significantly improved handwashing quality of our participants, regardless of their prior hand hygiene knowledge levels. Installing hand scanners in health care settings could help maintain high levels of hand hygiene compliance and practice, although this might be limited by cost and space constraints. A portable device could be a solution for auditing and training at the point of care.

Hand scan images may help nursing students identify overlooked handwashing areas. However, our study found minimal impact on handwashing effectiveness, with no significant differences in fluorescent residues between the hand scan image and control groups after the intervention. A previous study also found that fluorescent lotion alone was not enough to enhance handwashing effectiveness.^31^ It is therefore essential to integrate new techniques into training programs.

One notable finding from our study is that a 15-minute educational program significantly improved hand hygiene knowledge and handwashing quality among nursing students. This is consistent with the findings of previous studies that brief interventions in enhancing hand hygiene compliance and knowledge in health care workers and medical students.^32,33^ Given the success of our program, it can be easily integrated into students’ curricula and repeated as needed. However, it is important to note that maintaining and further improving hand hygiene practices require continuous training.^27^ While single educational sessions may lead to temporary improvements, a sustained and ongoing training approach is essential to reinforce good habits and keep students updated on the latest hand hygiene guidelines.

Interestingly, we observed that all participants increased their handwashing time during their second attempt, regardless of their group assignment. The intervention groups showed a greater increase in the total duration of the 6 or 7 handwashing steps compared with the control group. However, this prolonged handwashing time did not lead to a significantly greater reduction in fluorescent residues after the intervention compared with the control group. This discrepancy might be explained by our recent finding that the optimal overall handwashing time is 31 seconds from step 1 to step 7, and 28 seconds from step 1 to step 6, with each step ideally lasting 4 to 5 seconds.^22^ This suggests that the duration of each step should be integrated into hand hygiene education programs to prevent unnecessarily prolonged handwashing time, which does not improve performance or decontamination effects.

### Limitations

There were several limitations in our study. We only measured the immediate effects of our education program, but the long-term effects remain unexplored. Future research could involve follow-up data to investigate the sustainability of benefits over time. Our program only included first-year nursing students. Future studies may involve all health-related disciplines from multiple centers. Our research was conducted in a university setting where antiseptic soap was used for hand hygiene practices. However, in health care settings, alcohol-based hand rubs are more common.^34,35^ Further studies are needed to evaluate the program’s effect with alcohol-based hand rubbing. Although the exact amount of liquid soap for each participant was not rigidly controlled, our research assistants made an effort to ensure a rough consistency in soap volume by administering 1 pump of liquid soap onto the hands of each participant. While we did not separately assess the decontamination effect of our education program on the WHO 6-step and local 7-step techniques, the performance quality assessments had similar results for techniques.

## Conclusions

Our study demonstrated that a multifaceted program did not significantly increase the effect of decontamination among nursing students. However, the intervention did improve the understanding and performance quality of handwashing techniques.

## Notes

Supplement 1.
Trial Protocol

Supplement 2. **eMethods.** Supplementary Methods
eReferences.
**eTable 1.** Correct Performance and Duration of Hand Hygiene Steps **eTable 2.** Knowledge Before and After Intervention **eTable 3.** Attitude Before and After Intervention **eFigure.** Effect of Hand Hygiene Educational Interventions on Handwashing Duration and Percentage of Correctly Performed Steps

Supplement 3.
Data Sharing Statement
