# Supplementary material for: Hand Hygiene Education Components Among First-Year Nursing Students: A Cluster Randomized Clinical Trial
Source: JAMA Netw Open. 2024 Jun 13;7(6):e2413835. doi: 10.1001/jamanetworkopen.2024.13835 (PMC11177166; doi:10.1001/jamanetworkopen.2024.13835)
Supplement: Supplement 1. — Trial Protocol [file jamanetwopen-e2413835-s001.pdf]

Protocol

The effectiveness of a multicomponent education program of hand hygiene in nursing students: a randomized controlled trial

Background

The ongoing COVID-19 pandemic has posed serious threats to public health worldwide. Although children have mild symptoms, they have been found to more likely transmit the virus within the household than the other age groups. The importance of hand hygiene cannot be underestimated in both healthcare and community settings (Allegranzi et al., 2011; Mathur, 2011). The promotion of hand hygiene is the best practices in preventing health care-associated infection (WHO, 2009). It has been estimated that hand hygiene could reduce over 500 000 attributable deaths per year. It was found that the annual economic impact of health care-associated infection in the US was approximately US$6.5 billion in 2004 (WHO, 2009) and every US$1 spent on hand hygiene promotion could result in a US$23.7 benefit (Chen et al., 2011). Education plays a key role in setting a good practice base in hand hygiene.

The traditional training methods for nursing students include in-class lecture and practical, but students seldom receive individualized instructions and feedbacks due to limited manpower and facilities. Hence, there is an urgent need of designing an automated and individualized education program. This study aims to design and test a multicomponent education program of hand hygiene in nursing students. Specifically, this program has two components: 1) training videos according to the WHO guidelines on hand hygiene in health care (https://polyu.ap.panopto.com/Panopto/Pages/Viewer.aspx?id=f3973cee-bcfb-48fd-a306-b6893dacaabd); 2) a hand scanner used for students to immediately visualize the missing spots of hand hygiene.

This study has the following specific objectives:

1) conduct a randomized controlled trial (RCT) to test the effectiveness of this program on knowledge and practice of hand hygiene in nursing students;

2) evaluate the effects of each component and collect feedbacks from participants.

If demonstrated effective, this program can be integrated into the curriculum of nursing students as an e-learning approach. In future, this system can also be used at point-of-care for real-time monitoring and audit of healthcare workers in other healthcare settings.

Methods

Study design
This is a four-arm RCT.

Subject recruitment
We will recruit all year one nursing students from the Bachelor of Nursing and Bachelor of Mental Health Nursing program in School of Nursing (total number is approximately 320) during September to November 2022. The participants will subscribe a timeslot of training sessions (each session has 4 participants). All participants who attend the same session will be assigned to the same group by cluster randomization. A statistician will assign the groups in advance by a random number generator. The participants will not be informed about their group before arriving the study site (the Squina International Centre for Infection

Control in PolyU).

We calculated the statistical power for cluster randomized controlled trial. Since the total number is fixed to 320 and each cluster has 4 participants, there will be a total of 80 clusters which are equally divided into four groups. We assume the standard deviation of subjects is 2.00, the intracluster correlation coefficient is 0.010, and the coefficient of variation of cluster sizes is 0.500. The sample size of 320 can achieve 87% power to detect a difference between the group means of at least 1 in a two-sided t-test with a significance level of 0.050.

Pre-intervention assessment

During the site visit, the participants will sign a consent form first and be asked to fill in a questionnaire on knowledge and practice of hand hygiene (attached in Appendix).

Participants will need to put fluorescent powder on both of their hands and then perform hand washing using liquid soap without any instructions (1st HW attempt). The camera installed above the hand-wash basins will take videos of both hands (no face nor other parts of the body) during this procedure. After washing hands, participants will need to scan fluorescent powder remained on their hands in a hand scanner (The Semmelweis Scanner™). The recorded hand washing videos will be used to train the AI algorithms for automatic image processing and assessments. Two IPC experts will judge the quality of hand hygiene in these videos, which will be adopted as ground truth in image processing. The percentage of fluorescent gel residual on hands shown in the hand scanner will be used as an objective assessment for efficacy of hand washing in individual participants.

Intervention and control groups

Group A (Hand scanner instant feedback)

Participants will be informed about their hand scanner results of the first HW attempt to get individualized feedback about their hand wash performance. But no training video is provided.

Group B (Video training)

Participants will watch a training video about the 7 steps of hand washing after the first HW attempt, but they will not be informed about their hand scanner results.

Group C (Hand scanner + Video training)

Participants will be informed about their hand scanner results during the first HW attempt. They will aslo watch a training video about the 7 steps of hand washing.

Group D (Control group without intervention)

Participants will not be informed about the hand scanner results in the first HW attempt and no training videos will be provided.

Post-intervention assessment

The participants will take the second HW attempt by putting fluorescent powder on both of their hands again and then perform hand wash with videos recorded. After washing hands, participants will scan fluorescent powder remained on their hands again in a hand scanner.

Participants will also be asked to fill in a questionnaire on knowledge and practice of hand hygiene as part

of post-intervention assessment.

For the training purpose, after the completion of post-intervention measurements, the participants of the control group (Group D) will watch the training video and repeated hand washing and scanning for the third time if they agree. But the results of their third HW attempt will not be included into data analysis in this study.

Randomization, allocation concealment, and blinding

Participants will be randomly assigned to the intervention groups or control group through a process of randomization performed by a statistician who will not be involved in subject recruitment. The participants will be blinded. The RAs involved in subject recruitment and the IPC experts who judge the quality of hand hygiene will be blinded to the groupings. The RAs and student assistants involved in hand hygiene video recording at the study site will not be blinded because they will be giving instructions to the participants.

Ethical consideration

It is anticipated that the study will not pose any physical or psychological harm to the participants. The contact details of either the ethical or the research in-charge personnel are clearly written in the information sheet.

Explanation of the study will be given to participants who are interested in participating in the study. Participants will also be given the opportunity to ask our project personnel questions. Furthermore, the information sheet including the purpose of the study, confidentiality of the data, and rights of the participants, will also be provided to the participants. The contact details of either the ethical or the research in-charge personnel are clearly written in the information sheet. If they agree to participate in the study, they are required to sign the consent form. Their participation is on a voluntary basis, they have the right to withdraw from the study at any time if they want to without consequence.

All the collected data will be kept confidentially, and only the research personnel can access and handle the data. No personal information of the participants will be collected except the student ID number.

Confidentiality and Security

Data collection will be anonymous, only analysed for specified purposes and on the basis of the consent or other legitimate basis laid down by law. No personal information will be linked to image/video data. All the image/video data will be deleted one year after the project is completed. The Project will not involve any activities or results raising security issues.

References

Al-Shatri, H., Müller, S., & Klein, A. (2016, 22-27 May 2016). Distributed algorithm for energy efficient multi-hop computation offloading. 2016 IEEE International Conference on Communications (ICC),

Allegranzi, B., Bagheri Nejad, S., Combescure, C., Graafmans, W., Attar, H., Donaldson, L., & Pittet, D. (2011). Burden of endemic health-care-associated infection in developing countries: systematic

review and meta-analysis. *Lancet*, *377*(9761), 228-241. https://doi.org/10.1016/S0140-6736(10)61458-4

Chen, Y. C., Sheng, W. H., Wang, J. T., Chang, S. C., Lin, H. C., Tien, K. L., Hsu, L. Y., & Tsai, K. S. (2011). Effectiveness and limitations of hand hygiene promotion on decreasing healthcare-associated infections. *PLoS One*, *6*(11), e27163. https://doi.org/10.1371/journal.pone.0027163

Gasteiger, N., Dowding, D., Ali, S. M., Scott, A. J. S., Wilson, P., & van der Veer, S. N. (2021). Sticky apps, not sticky hands: A systematic review and content synthesis of hand hygiene mobile apps. *J Am Med Inform Assoc*, *28*(9), 2027-2038. https://doi.org/10.1093/jamia/ocab094

Mathur, P. (2011). Hand hygiene: back to the basics of infection control. *Indian J Med Res*, *134*(5), 611-620. https://doi.org/10.4103/0971-5916.90985

Mueller, F., Bernard, F., Sotnychenko, O., Mehta, D., Sridhar, S., Casas, D., & Theobalt, C. (2018). GANerated Hands for Real-time 3D Hand Tracking from Monocular RGB. *In Proceedings of the IEEE Conference on Computer Vision and Pattern Recognition*, 49-59.

WHO. (2009). In *WHO Guidelines on Hand Hygiene in Health Care: First Global Patient Safety Challenge Clean Care Is Safer Care*. https://www.ncbi.nlm.nih.gov/pubmed/23805438

Yang, L., Lu, Y., Cao, J., Huang, J., & Zhang, M. (2020). E-Tree Learning: A Novel Decentralized Model Learning Framework for Edge AI. *IEEE Internet of Things Journal*.

Protocol

Appendix

Survey on knowledge and practice of Hand Hygiene in Nursing Students

Part I: Demographic

1. Student ID: ______________________
2. Age: ______________________ yrs
3. Gender: Female Male
4. Programme: BSN Mental Health Nursing

Part II: Hand hygiene

5. Have you ever received formal training in hand hygiene?
   Yes No
   If your answer is yes; when did you receive the training (give the latest year)?
   Answer: ___________________(yyyy)

6. How many steps are required for healthcare workers to perform hand hygiene (please give a number) ?
   Answer: ______________________

7. How many types of hand hygiene are there (please give a number)?
   Answer: ______________________

8. How long is the minimal duration of hand washing?
   Answer: ______________________ seconds

9: Which of the following sequence is correct for hand hygiene? (MC Question)

A	B	C
Finger webs	Finger webs	Thumbs
Finger tips	Back of fingers	Finger tips
Back of fingers	Thumbs	Finger webs
Thumbs	Finger tips	Back of fingers

10. Which of the following is one of hand hygiene steps EXCEPT?
    Rub palms
    Rub fingernails
    Rub wrists
    Fingers interlocked

11. Do you agree or disagree with the following statement:
    Hand hygiene is part of infection prevention and control in healthcare settings.
    Strongly agree
    Agree
    Neither agree nor disagree
    Disagree
    Strongly disagree

Additional questions for post evaluation

12. What are the 7-steps of hand washing? (MC Questions)

	A	B	C
Step 1	Finger tips	Palms	Between fingers
Step 2	Back of hands	Back of fingers	Palms
Step 3	Thumbs	Finger tips	Between fingers
Step 4	Palms	Between fingers	Back of hands
Step 5	Finger tips	Thumbs	Back of fingers
Step 6	Back of fingers	Finger tips	Wrists
Step 7	Wrists	Thumbs	Back of hands

13. What is the sequence for hand hygiene?

Steps			Drag Answer Here:
Finger tips		Step 1	
Back of hands		Step 2	
Thumbs		Step 3	
Palms		Step 4	
Finger tips		Step 5	
Back of fingers		Step 6	
Wrists		Step 7	
