# Supplementary material for: Hand Hygiene Education Components Among First-Year Nursing Students: A Cluster Randomized Clinical Trial
Source: JAMA Netw Open. 2024 Jun 13;7(6):e2413835. doi: 10.1001/jamanetworkopen.2024.13835 (PMC11177166; doi:10.1001/jamanetworkopen.2024.13835)
Supplement: Supplement 2. — eMethods. Supplementary Methods eReferences. eTable 1. Correct Performance and Duration of Hand Hygiene Steps eTable 2. Knowledge Before and After Intervention eTable 3. Attitude Before and After Intervention eFigure. Effect of Hand Hygiene Educational Interventions on Handwashing Duration and Percentage of Correctly Performed Steps [file jamanetwopen-e2413835-s002.pdf]

Supplemental Online Content

Chen J, Yang L, Mak Y, et al. Hand hygiene education components among first-year nursing students: a cluster randomized clinical trial. *JAMA Netw Open.* 2024;7(6):e2413835. doi:10.1001/jamanetworkopen.2024.13835

eMethods. Supplementary Methods

eReferences.

eTable 1. Correct Performance and Duration of Hand Hygiene Steps

eTable 2. Knowledge Before and After Intervention

eTable 3. Attitude Before and After Intervention

eFigure. Effect of Hand Hygiene Educational Interventions on Handwashing Duration and Percentage of Correctly Performed Steps

This supplemental material has been provided by the authors to give readers additional information about their work.

© 2024 Chen J et al. *JAMA Network Open*.

eMethods. Supplementary Methods

Data collection

Before the intervention, participants were required to fill out a questionnaire to provide information about their socio-demographics and previous hand hygiene training. They were then asked to apply a sufficient amount of fluorescent lotion (*GlitterBug™, Brevis Corporation, USA*) to their hands, ensuring complete coverage. A "Hand-in-Scan" Semmelweis Hand Hygiene Scanner (*HandInScan Zrt. Debrecen Hungary. Model: HINST20E3WS0P01*) was used to confirm that their hands and wrists were completely covered with the fluorescent lotion. This hand scanner has been validated in previous studies for its effectiveness in assessing hand hygiene.[1, 2]

In our study, participants washed their hands with liquid soap and water. After scanning their hands, a research assistant guided them through the handwashing process, which was recorded on camera. Participants in each cluster were randomly assigned to one of four groups: (1) *Hand scan image* group, who reviewed the hand scan images and received immediate feedback and explanations about their handwashing techniques; (2) *Instructional video* group, who watched a training video demonstrating the seven steps of handwashing according to the local guideline[25] (see the eAppendix in Supplement 2). The local guideline mirrored the WHO 6-step technique, with the addition of a 7th step - rubbing wrists to the end. Therefore, our evaluation encompassed both the WHO 6-step and the local 7-step techniques.; (3) *Hand*

© 2024 Chen J et al. *JAMA Network Open*.

scan image + Instructional video group, who received visual feedback from their first handwashing attempt and watched the instructional video; (4) *Control* group, where participants did not receive feedback or watch the instructional video. The interventions were given after their first attempt before proceeding to their second attempt. For training purposes, the control group watched the instructional video after the post-intervention handwashing measurements. If they consented, they repeated the handwashing and scanning process for a third time, but their results from the third attempt were excluded from data analysis.

Each participant was instructed to perform two handwashing attempts. They were not given any instructions on handwashing techniques before their first attempt. After the first attempt, participants scanned their hands again to check for any remaining fluorescent residue. Depending on their assigned group, participants received immediate feedback from the hand scanner, watched an instructional video on the 7-step handwashing techniques, or both, before repeating the handwashing process for the second attempt. An RGB camera positioned above the handwash basin recorded participants' hand movements during handwashing, excluding their faces. The quality of hand hygiene performance in the videos was evaluated independently by two experts in infection prevention and control, considering the accuracy and duration of each of the seven steps.

© 2024 Chen J et al. *JAMA Network Open*.

eReferences.

1. Lehotsky A, Szilagyi L, Bansaghi S, Szeremy P, Weber G, Haidegger T. Towards objective hand hygiene technique assessment: validation of the ultraviolet-dye-based hand-rubbing quality assessment procedure. J Hosp Infect. 2017;97(1):26-9.

2. Bansaghi S, Sari V, Szeremy P, Lehotsky A, Takacs B, Toth BK, et al. Evidence-based Hand Hygiene - Can You Trust the Fluorescent-based Assessment Methods? Acta Polytech Hung. 2021;18(11):269-83.

© 2024 Chen J et al. *JAMA Network Open*.

eTable 1. Correct Performance and Duration of Hand Hygiene Steps

	Control	Hand scan image	Instructional video	Hand scan image + Instructional video	ICC (SE) #	95%CI
	**n=66**	**n=68**	**n=67**	**n=69**		
**Step duration (seconds)**						
**All 7 steps**						
Before intervention	46 (39, 52)	35 (28, 41)	36 (29, 42)	35 (29, 41)	0.02 (0.03)	0.0-0.08
After intervention	52 (46, 58)	49 (43, 55)	50 (44, 56)	51 (45, 57)	0.0 (0.01)	0.0-0.02
p-value##		**.001**	**.002**	**<.001**		
**All 6 steps**						
Before intervention	38 (32, 43)	29 (23, 34)	29 (24, 35)	29 (24, 35)	0.01 (0.02)	0.0-0.06
After intervention	43 (38, 48)	41 (36, 46)	42 (37, 47)	43 (38, 48)	0.0 (0.01)	0.0-0.02
p-value##		**.003**	**.003**	**.001**		
**Step 1 only**						
Before intervention	5 (4 ,6)	3 (2 ,4)	3 (2 ,4)	4 (3 ,5)	0.03 (0.04)	0.0-0.11
After intervention	5 (4 ,6)	5 (4 ,6)	4 (3 ,5)	5 (4 ,6)	0.0 (0.01)	0.0-0.02
p-value##		**.003**	.16	.10		
**Step 2 only**						
Before intervention	9 (7 ,10)	6 (5 ,8)	7 (5 ,8)	7 (5 ,8)	0.01 (0.02)	0.0-0.04
After intervention	10 (8 ,12)	11 (9 ,13)	10 (8 ,11)	11 (9 ,12)	0.0 (0.01)	0.0-0.02
p-value##		**.001**	.12	**.005**		
**Step 3 only**						
Before intervention	6 (5 ,7)	5 (3 ,6)	4 (3 ,6)	5 (4 ,6)	0.001 (0.01)	0.0-0.03
After intervention	6 (5 ,8)	7 (6 ,8)	6 (5 ,7)	5 (4 ,6)	0.02 (0.02)	0.0-0.06
p-value##		**.03**	.31	.52		
**Step 4 only**						
Before intervention	6 (4 ,7)	5 (4 ,6)	4 (2 ,5)	4 (3 ,6)	0.01 (0.02)	0.0-0.04
After intervention	7 (6 ,9)	6 (5 ,8)	7 (5 ,8)	7 (5 ,8)	0.0 (0.01)	0.0-0.02
p-value##		.84	.16	.34		
**Step 5 only**						
Before intervention	6 (4 ,7)	4 (3 ,5)	5 (4 ,6)	5 (3 ,6)	0.004 (0.02)	0.0-0.03
After intervention	6 (5 ,7)	5 (4 ,7)	7 (6 ,8)	7 (6 ,8)	0.01 (0.02)	0.0-0.06
p-value##		.16	**.02**	**<.001**		

© 2024 Chen J et al. *JAMA Network Open*.

	Control	Hand scan image	Instructional video	Hand scan image + Instructional video	ICC (SE) #	95%CI
**Step 6 only**						
Before intervention	7 (6 ,8)	6 (4 ,7)	6 (5 ,7)	5 (4 ,6)	0.01 (0.02)	0.0-0.05
After intervention	8 (7 ,9)	6 (5 ,8)	8 (7 ,10)	8 (7 ,9)	0.01 (0.02)	0.0-0.05
p-value##		.53	.37	.06		
**Step 7 only**						
Before intervention	8 (7 ,9)	6 (4 ,7)	6 (5 ,8)	6 (4 ,7)	0.03 (0.03)	0.0-0.09
After intervention	9 (8 ,10)	8 (7 ,9)	8 (7 ,10)	8 (7 ,10)	0.0 (0.01)	0.0-0.02
p-value##		.07	.11	**.006**		
**Percentage of correctly performed steps**						
**All 7 steps**						
Before intervention	18.0 (8.6, 27.4)	3.0 (-5.5, 11.4)	10.5 (2.2, 18.9)	14.6 (6.3, 22.8)	0.03 (0.03)	0.0-0.09
After intervention	19.6 (8.4, 30.7)	5.9 (-4.2, 16.0)	32.9 (22.9, 42.9)	20.4 (10.4, 30.3)	0.06 (0.06)	0.0-0.17
p-value##		.83	**.002**	.51		
**All 6 steps**						
Before intervention	18.1 (8.8, 27.3)	4.4 (-4.0, 12.8)	10.5 (2.2, 18.9)	14.5 (6.3, 22.8)	0.02 (0.03)	0.0-0.07
After intervention	19.6 (8.6, 30.5)	5.9 (-4.1, 15.8)	32.9 (23.0, 42.8)	20.3 (10.5, 30.2)	0.06 (0.06)	0.0-0.17
p-value##		>.99	**.001**	.51		
**Step 1 only**						
Before intervention	83.3 (72.2, 94.3)	82.1 (71.2, 92.9)	80.6 (69.9, 91.2)	82.7 (72.1, 93.3)	0.0 (0.01)	0.0-0.02
After intervention	84.1 (74.1, 94.0)	85.0 (75.3, 94.7)	89.5 (80.0, 99.0)	94.3 (84.9, 103.8)	0.004 (0.02)	0.0-0.04
p-value##		.76	.42	.18		
**Step 2 only**						
Before intervention	53.0 (38.1, 67.9)	22.1 (10.7, 33.4)	43.3 (31.9, 54.7)	43.5 (32.2, 54.7)	0.05 (0.05)	0.0-0.16
After intervention	50.0 (35.0, 64.9)	26.5 (15.4, 37.6)	70.2 (59.0, 81.3)	68.1 (57.1, 79.1)	0.15 (0.11)	0.0-0.37
p-value##		.38	**<.001**	**.002**		
**Step 3 only**						
Before intervention	42.5 (29.1, 55.9)	43.9 (31.6, 56.2)	40.3 (28.0, 52.6)	59.6 (47.5, 71.8)	0.02 (0.02)	0.0-0.06
After intervention	51.6 (38.3, 64.9)	39.5 (27.5, 51.6)	67.2 (55.1, 79.2)	65.4 (53.5, 77.4)	0.05 (0.05)	0.0-0.15
p-value##		.18	.10	.64		

	Control	Hand scan image	Instructional video	Hand scan image + Instructional video	ICC (SE) [#]	95%CI
**Step 4 only**						
Before intervention	30.1 (17.7, 42.6)	22.1 (11.3, 32.8)	32.8 (22.0, 43.7)	31.9 (21.2, 42.6)	0.0 (0.10)	0.0-0.20
After intervention	30.1 (16.9, 43.3)	23.5 (12.5, 34.5)	65.7 (54.6, 76.8)	59.4 (48.5, 70.4)	0.16 (0.12)	0.0-0.25
p-value[##]		.84	**<.001**	**<.001**		
**Step 5 only**						
Before intervention	52.7 (38.5, 66.9)	39.7 (27.1, 52.2)	50.7 (38.2, 63.2)	45.0 (32.6, 57.3)	0.0 (0.01)	0.0-0.02
After intervention	54.2 (40.5, 67.9)	45.5 (33.8, 57.2)	76.1 (64.4, 87.8)	79.7 (68.2, 91.3)	0.10 (0.08)	0.0-0.27
p-value[##]		.62	**.01**	**<.001**		
**Step 6 only**						
Before intervention	72.7 (60.6, 84.8)	55.9 (44.4, 67.4)	59.7 (48.1, 71.3)	52.2 (40.8, 63.6)	0.02 (0.03)	0.0-0.07
After intervention	70.4 (58.9, 81.9)	52.9 (42.3, 63.6)	82.1 (71.4, 92.8)	73.9 (63.4, 84.4)	0.06 (0.05)	0.0-0.16
p-value[##]		.95	**.008**	**.009**		
**Step 7 only**						
Before intervention	68.1 (54.1, 82.1)	67.5 (55.7, 79.2)	71.6 (59.8, 83.3)	69.6 (58.0, 81.2)	0.0 (0.01)	0.0-0.02
After intervention	81.7 (69.9, 93.6)	63.1 (54.0, 72.1)	96.9 (88.0, 105.9)	95.7 (86.8, 104.6)	0.17 (0.12)	0.0-0.41
p-value[##]		**.02**	.19	.16		

Data presented as mean (95%CI), estimations using margins command in STATA post mixed model with cluster as the fixed effect.

[#] Negative values have been truncated by loneway command in STATA

[##] Difference in changes pre- and post-intervention, each intervention group compared to control; Model adjusted for baseline age, sex, program attended, previous hand hygiene training experience and pre-intervention measures

© 2024 Chen J et al. *JAMA Network Open*.

eTable 2. Knowledge Before and After Intervention

	Control	Hand scan image	Instructional video	Hand scan image + Instructional video	ICC (SE)[#]	95%CI
	**n=66**	**n=68**	**n=67**	**n=69**		
**7 step hand hygiene technique**						
Before intervention	32 (48.5)	35 (51.5)	37 (56.1)	40 (58.8)	0 (0.03)	0.0-0.06
After intervention	37 (56.1)	37 (55.2)	47 (70.1)	46 (66.7)	0 (0.03)	0.0-0.06
p-value[##]		.63	.40	.86		
**Types of hand hygiene**						
Before intervention	33 (50.0)	35 (51.5)	33 (50.0)	31 (45.6)	0 (0.01)	0.0-0.02
After intervention	34 (51.5)	34 (50.7)	24 (35.8)	27 (39.1)	0.04 (0.04)	0.0-0.13
p-value[##]		>.99	**<.001**	.13		
**Minimum duration of hand hygiene**						
Before intervention	32 (48.5)	33 (48.5)	21 (31.8)	38 (55.9)	0.03 (0.04)	0.0-0.10
After intervention	30 (45.5)	27 (40.3)	20 (29.9)	34 (50.7)	0 (0.03)	0.0-0.06
p-value[##]		.46	.81	.65		
**Sequence of hand hygiene steps**						
Before intervention	42 (63.6)	48 (70.6)	38 (57.6)	41 (60.3)	0 (0.03)	0.0-0.06
After intervention	50 (75.8)	48 (71.6)	51 (76.1)	53 (76.8)	0 (0.03)	0.0-0.06
p-value[##]		.20	.38	.64		
**Unclassified step**						
Before intervention	25 (37.9)	38 (55.9)	26 (39.4)	34 (50.0)	0.04 (0.04)	0.0-0.12
After intervention	26 (39.4)	41 (61.2)	45 (67.2)	45 (65.2)	0.08 (0.05)	0.0-0.17
p-value[##]		.70	**.004**	.14		

Data presented as n (%). Before and after difference was tested by McNemar test and Wilcoxon Signed Rank test as appropriate.

[#] Negative values have been truncated by loneway command in STATA

[##] Difference in changes pre- and post-intervention, each intervention group compared to control; Model adjusted for baseline age, sex, program attended, previous hand hygiene training experience and pre-intervention measures

© 2024 Chen J et al. *JAMA Network Open*.

eTable 3. Attitude Before and After Intervention

	Control	Hand scan image	Instructional video	Hand scan image + Instructional video	ICC (SE)[#]	95%CI
	**n=66**	**n=68**	**n=67**	**n=69**		
**Hand hygiene is part of infection prevention and control in healthcare settings [** Answer = Strongly agree]						
Before intervention	52 (78.8)	51 (75.0)	46 (69.7)	55 (80.9)	0.006 (0.03)	0.0-0.07
After intervention	56 (84.8)	57 (85.1)	54 (80.6)	62 (89.9)	0.05 (0.04)	0.0-0.13
p-value[##]		.51	.38	.59		

Data presented as n (%). Before and after difference was tested by McNemar test and Wilcoxon Signed Rank test as appropriate.

[#] Negative values have been truncated by loneway command in STATA

[##] Difference in changes pre- and post-intervention, each intervention group compared to control; Model adjusted for baseline age, sex, program attended, previous hand hygiene training experience and pre-intervention measures

© 2024 Chen J et al. *JAMA Network Open*.

eFigure. Effect of Hand Hygiene Educational Interventions on Handwashing Duration and Percentage of Correctly Performed Steps

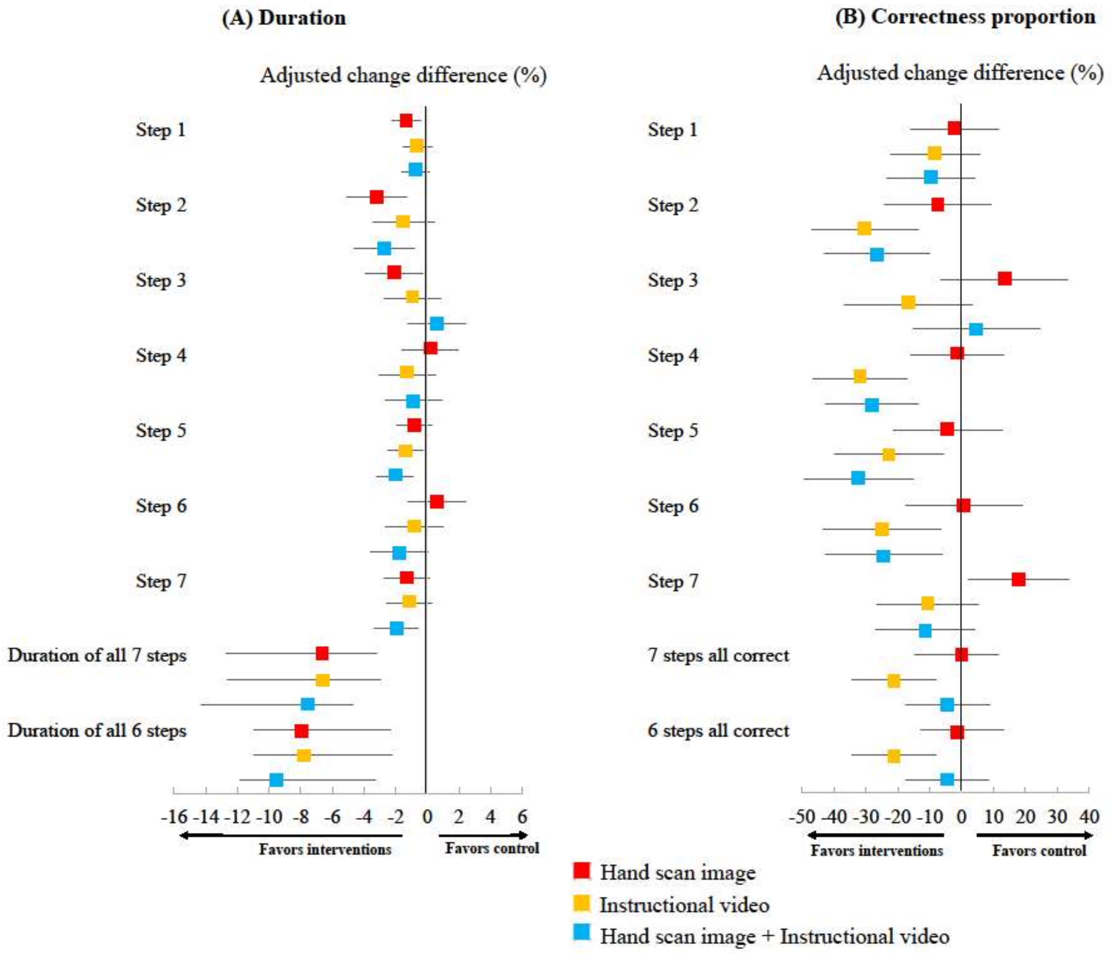

© 2024 Chen J et al. *JAMA Network Open*.
