# Supplementary material for: Hand Hygiene Education Components Among First-Year Nursing Students: A Cluster Randomized Clinical Trial
Source: JAMA Netw Open. 2024 Jun 13;7(6):e2413835. doi: 10.1001/jamanetworkopen.2024.13835 (PMC11177166; doi:10.1001/jamanetworkopen.2024.13835)
Supplement: Supplement 3. — Data Sharing Statement [file jamanetwopen-e2413835-s003.pdf]

Data Sharing Statement

Chen. Hand Hygiene Education Components Among First-Year Nursing Students. *JAMA Netw Open*. Published June 13, 2024. doi:10.1001/jamanetworkopen.2024.13835

Data
Data available: No

Additional Information
Explanation for why data not available: All data will be shared upon request to the corresponding author.
